# DT-13 Mediates Ligand-Dependent Activation of PPARγ Response Elements In Vitro

**DOI:** 10.3390/biology13121015

**Published:** 2024-12-04

**Authors:** Shikha Raina, Esther Samuel, Hendrik Fuchs

**Affiliations:** Charité—Universitätsmedizin Berlin, Corporate Member of Freie Universität Berlin and Humboldt-Universität zu Berlin, Institute of Diagnostic Laboratory Medicine, Clinical Chemistry and Pathobiochemistry, Augustenburger Platz 1, D-13353 Berlin, Germany

**Keywords:** saponin, anti-inflammation, peroxisome proliferator-activated receptor gamma, transrepression, nuclear factor kappa B

## Abstract

Activation of inflammatory pathways releases a storm of cytokines. Moreover, unregulated cytokines contribute to chronic inflammatory disorders. However, ligand-activated peroxisome proliferator-activated receptor gamma (PPARγ) is involved in suppressing inflammatory cytokines via transrepression of nuclear factor kappa B (NFκB). Therefore, in this study, the anti-inflammatory saponin DT-13 is explored as a ligand of PPARγ. DT-13 upregulated the expression of PPARγ in lipopolysaccharide (LPS)-stimulated RAW264.7 cells in comparison to treatment with LPS alone. Applying a HEK transfection model, we observed a DT-13 dose-dependent increase in ligand-dependent activation of PPARγ, which was compared with troglitazone and rosiglitazone. DT-13 was not able to compete with the synthetic fluoromone tracer for binding to PPARγ as observed in a fluorescence polarization binding assay, whereas molecular docking showed a possible binding interaction of DT-13 with the PPARγ nuclear receptor. We proved the expression of PPARγ protein in the presence of DT-13 using a robust cell-based HEK293FT transfection model. More in-depth analysis needs to be performed to evaluate the efficiency of the binding of DT-13 to PPARγ. A possible binding interaction of DT-13 to PPARγ was observed, similar to that of rosiglitazone. This study revealed a novel mechanism for anti-inflammatory effects by DT-13 through PPARγ-dependent transrepression of NFκB.

## Simple Summary

Inflammation poses one of the causes for progressing into the chronic stages of a disease. Therefore, it is important to find the molecules that modulate the pathways involved in inflammation. Ancient medicinal drugs involving plant substances called saponins have been recently explored as anti-inflammatory drug candidates to ameliorate inflammation. Our aim was to find the connection between the saponin DT-13 and the nuclear receptor involved in inhibiting inflammation-related genes. We found that DT-13 is involved in the activation of peroxisome proliferator-activated receptor (PPARγ) in vitro to further regulate the expression of inflammatory genes. We also showed that DT-13 binds to the critical amino acid residue in PPARγ similar to that of the known anti-inflammatory and anti-diabetic drug rosiglitazone. Our results are important for further studies to develop anti-inflammatory drug candidates based on saponin DT-13.

## 1. Introduction

Inflammation is the natural response of the body to external stimuli like infection or injury. It activates various complex metabolic pathways that are meant to clear the cause of injury and further begin the tissue repair process [1]. There are two types of inflammation—acute and chronic. Acute inflammation is generally localized, protecting the body against harmful infections, and resolves after the damage is repaired [2], whereas chronic inflammation occurs due to persistent inflammation and forms the root cause of various detrimental diseases such as autoimmune diseases and chronic inflammatory diseases [3]. Inflammatory stimuli like bacterial endotoxin LPS are recognized by pattern recognition receptors (PRRs), for instance, by toll-like receptors (TLRs) present on the surface of macrophages, which further activates the inflammatory pathways [4]. In LPS-stimulated macrophages, the TLR4 signaling pathway is activated, leading to downstream regulation of signaling molecules via activation of NFκB to achieve a cytokine storm in the cells associated with inflammation [5]. The major component of the LPS-TLR4 pathway is the transcription factor NFκB that, upon activation and translocation into the nucleus, regulates the transcription of the proinflammatory genes responsible for the production of cytokines and other proteins associated with the inflammation [6]. Plant-derived glycolipids or glycosides, such as saponins, e.g., ginsenosides, are known to attenuate inflammation via inhibition of LPS-stimulated NFκB activation in macrophages [7,8]. Saponins have been widely studied as promising anti-inflammatory molecules for the development of plant-derived drugs [9,10,11]. In addition to their anti-inflammatory nature, these plant metabolites are known for their anti-cancer [12], anti-fungal [13] and anti-inflammasome [14] properties. This study aims to explore the anti-inflammatory properties of DT-13, a steroidal saponin obtained from the roots of *Liriope muscari* L. H. Bailey. DT-13 has been explored for its anti-inflammatory [15], anti-cancer [16] and cardioprotective [17] properties. In our previous studies, we have shown that DT-13 inhibits translocalization of activated NFκB into the nucleus and therefore downregulates the LPS-induced inflammatory cytokine production. We have also shown DT-13 as an attenuator of the NLRP3 inflammasome by regulating the expression of caspase 1, IL-1β and the NLRP3 gene [18].

In addition to the secondary metabolites of the TLR signaling pathway, ligand-activated nuclear receptors are also well known to play a role in regulating the immune responses via controlled gene expression. Huang and Glass summarized the roles of the glucocorticoid receptor (GR), peroxisome proliferator-activated receptors (PPARs), liver X receptors (LXRs) and nuclear receptor-related 1 protein (Nurr1) in transrepressing the inflammatory genes [19]. Members of nuclear receptors are involved in regulating various processes, including development and immunity, by interacting with the response elements on the respective target genes. The binding to response elements further initiates the recruitment of core activator complexes in a ligand-dependent manner [20]. The association of activator or repressor molecules decides the fate of the gene transcription mediated by nuclear receptors.

PPARγ is known to regulate the expression of proinflammatory genes and thereby is involved in modulating the response of macrophages to external stimuli [21]. In macrophages, upon pathogen stimulation, PPARγ in complex with antagonist molecules maintains the activation of NFκB and therefore upregulates the proinflammatory cytokine production [22,23]. Upon ligand activation of PPARγ, the corepressor molecules are removed, leading to inhibition of NFκB-mediated gene transcription. This results in an anti-inflammatory state called transrepression [24]. This phenomenon is exploited as the basis for the development of targeted drug therapeutics [25]. Lipids, glycolipids and cholesterol bind to PPARγ and activate the indirect inhibition of inflammatory pathways. Recently, members of nuclear receptors were investigated in silico to have binding pockets for saponins [26]. Wang et al. described natural products depicted as agonists of PPARγ and also focused on the methodologies used to screen the ligands [27]. So far there are no preclinical studies performed to confirm the association of DT-13 with nuclear receptors. In this study, our aim is to decipher the interaction of DT-13 as a ligand for PPARγ and explore PPARγ as a therapeutic target for the steroidal saponin DT-13 to further understand its anti-inflammatory mechanism.

## 2. Materials and Methods

Chemicals: Ginsenoside Rk1 (Merck, Darmstadt, Germany), *Liriope muscari* L. H. Bailey saponin C (Biosynth-Carbosynth, Staad, Switzerland), dimethyl sulfoxide (DMSO) (Sigma, St. Louis, MI, USA), lipopolysaccharide from *Escherichia coli* O111:B4 (Sigma, St. Louis, MI, USA), Dulbecco’s modified eagle medium (DMEM) (GIBCO by Life Technologies, Carlsbad, CA, USA), 0.25% trypsin-EDTA (GIBCO by Life Technologies, Carlsbad, CA, USA), fetal bovine serum (FBS) (Thermo Fisher Scientific, Schwerte, Germany), penicillin–streptomycin (Merck, Darmstadt, Germany).

Cell culture: RAW264.7 mouse macrophages were purchased from ATCC (Manassas, VA, USA), and HEK293 FT cells were obtained from Invitrogen^TM^ R70007 (Carlsbad, CA, USA). Cells were grown in DMEM supplemented with 10% FBS and 1% penicillin–streptomycin at 37 °C.

Western blotting: RAW264.7 cells were maintained in the complete media until 80% confluency. Cells were split into 100 mm dishes for Western blot protein analysis. For pre-treatment, cells were incubated with the compounds (Rk1, DT-13, dexamethasone and DMSO) for 1 h, followed by the addition of LPS (10 ng) without changing media for a total of 19 h. Cells were pelleted, and protein was extracted using radioimmunoprecipitation assay (RIPA)-lysis buffer. Each test sample (60 µg) was subjected to SDS-PAGE, and the protein was blotted onto a nitrocellulose membrane. Blocking buffer (5% BSA), PPARγ polyclonal primary antibody (1:1000) (#16643-1-AP, Proteintech, Germany) and 1:2000 secondary antibody (DAKO, Jena, Germany) were used.

Transient transfection: HEK293FT cells were used to transiently transfect three plasmids—pcDNA flag PPAR gamma (Addgene Plasmid #8895), PPRE X3-TK-luc (Addgene Plasmid #1015) and hRluc/SV40 (Renilla luciferase from Promega). Plasmids (100 ng pcDNA flag PPAR-gamma, 150 ng PPRE X3-TK-luc and 0.5 ng of Renilla luciferase pGL4.73 [hRluc/SV40]) were added in 25 µL Opti-MEM media in a 1.5 µL tube. In another tube, 1.5 µL lipofectamine was mixed with 25 µL Opti-MEM media. Both the tubes were mixed, and a total of 50 µL of the mixture was incubated for 15 min at room temperature and labeled as DNA:Lipofectamine mix. Another 200 µL DMEM was added on top of the DNA:Lipofectamine mix and kept for incubation for another 15 min at room temperature. Meanwhile, 250 µL of the medium was added in each well of a 96-well plate. The DNA:Lipofectamine mix was added slowly, dropwise, with a small pipette on top of each well. The plate was kept for incubation at 37 °C for 14–16 h. The next day, the transfection medium was replaced with complete media containing the test compounds (DT-13, Rk1, DMSO, troglitazone, rosiglitazone, SO1861). After 4–6 h of incubation, luciferase activity was measured using the Dual-Glo luciferase assay from Promega, following the manufacturer’s protocol.

Polar screen PPARγ green competitive assay: The binding efficiency of PPARγ ligands was compared using the PolarScreen™ PPARγ-Competitor Assay Kit, Green, from ThermoFisher Scientific. The assay includes a human-derived PPARγ ligand-binding domain (PPARγ-LBD) tagged with a GST tag and a selective green fluoromone (fluorescent PPARγ ligand) called PPARγ green complex. The assay is based on the competitive binding of the ligands by displacing the fluoromone from the PPARγ-LBD–fluoromone complex. The fluorescence polarization of the PPARγ green complex is high when compared to displaced fluoromone by the addition of ligands. This shift in polarization is used to evaluate the binding affinity of test compounds to PPARγ-LBD. The assay was carried out following the manufacturer’s protocol. The fluorescence polarization values expressed as millipolarization (mP) were measured at a wavelength of 485/535 nm for excitation/emission using a Tecan SPARK spectrofluorometer kindly provided by the SupraFAB lab, Freie Universität Berlin. The concentration at half-maximal shift in mP was defined as IC50 values. A curve-fitting wizard in GraphPad Prism 9.0. software was used to generate the IC50 values.

Molecular Docking: AutoDock methodology on Autodock tools 1.4.2. was used for molecular docking. The X-ray crystal coordinates of PPARγ in complex with rosiglitazone (PDB ID: 7AWC) with a resolution of 1.74 Å were used to perform the docking calculations. The pdbqt files for both DT13 and the ligand-free protein structure were generated for docking. The grid box was generated by selecting the grid center based on the bound ligand rosiglitazone, and the grid size was set to 60 × 60 × 60 xyz points with a grid spacing of 0.375 Å. The Lamarckian genetic algorithm available in Autodock was used to perform flexible docking between DT13 and PPARγ with the least binding energy. The representative figures of binding interactions were generated using BIOVIA Discovery Studio Visualizer.

Statistical analysis: GraphPad Prism 9.0. was used to analyze and plot the graphs of the experiments. A non-parametric Student t-test with Welch’s correction was used. All experiments were normalized, and outliers were removed using Z-score. The number of replicates is mentioned in the graphical representations separately.

## 3. Results

### 3.1. Saponins as an Inducer of PPARγ in LPS-Activated RAW Macrophages

**Figure A1:**
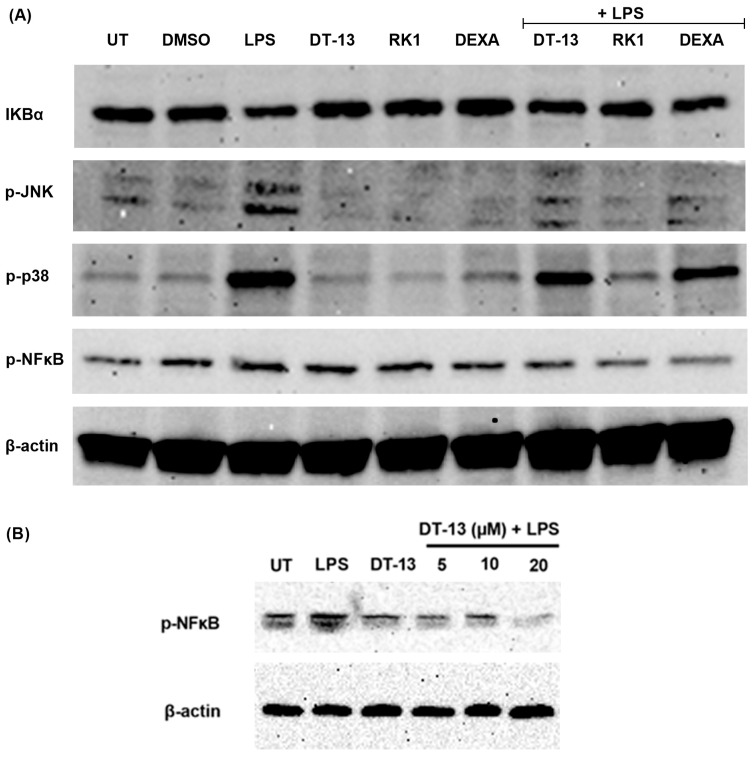
Protein expression of NFκB signaling proteins in LPS-stimulated RAW264.7 macrophages in the presence of DT-13: (**A**) Western blot analysis of p-JNK, p-NFκB, p-P38 MAPK and IKBα in the presence of DT-13-, Rk1- and dexamethasone (Dexa)-pretreated LPS-stimulated cells; (**B**) a dose-dependent decrease in p-NFκB protein expression in DT-13 pretreatments in LPS-stimulated cells. The protein expression is normalized using β-actin as an internal control. The Western blot image is representative of three independent experiments.

To investigate the inhibition of NFκB protein activation by DT-13, we checked the expression of phosphorylated proteins in LPS-stimulated RAW264.7 pretreated with DT-13. We observed a reduction in protein expression of phosphorylated NFκB (p-NFκB) (Appendix A Figure A1). This confirmed our previous findings that DT-13 attenuates inflammation via interfering with the LPS/TLR4/NFκB axis and especially inhibited the localization of NFκB into the nucleus as shown by immunofluorescence [18]. To further understand the effect of DT-13 on the expression of PPARγ in LPS-stimulated RAW264.7 macrophages, a Western blot analysis was performed. RAW264.7 cells were treated with dexamethasone (a known anti-inflammatory drug) and saponins (DT-13, Rk1). For stimulation, 10 ng of LPS was added. Further, total cell protein was used to analyze the expression of PPARγ.

**Figure 1:**
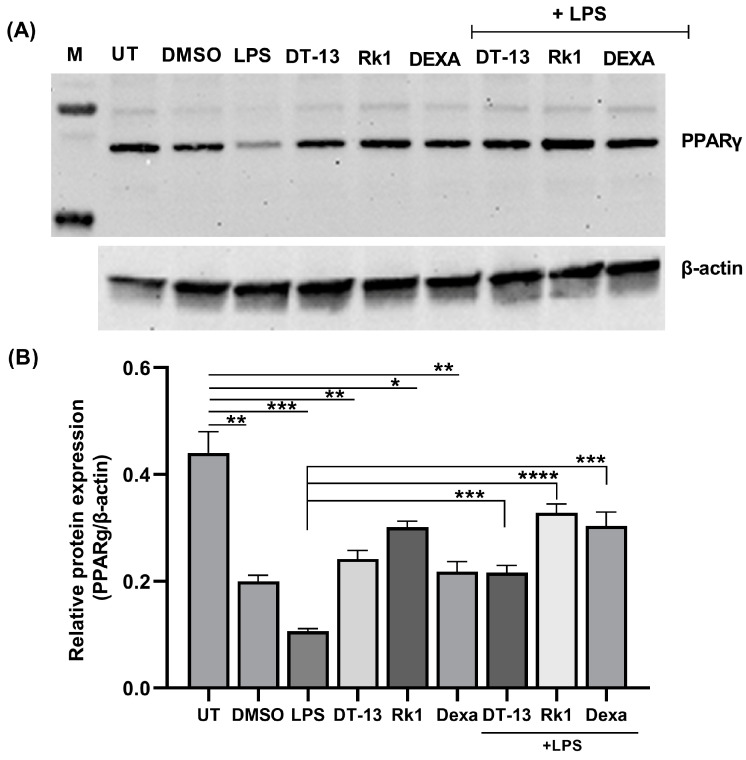
Saponins as an inducer of PPARγ in LPS-activated RAW macrophages: (**A**) representative image of a Western blot against PPARγ protein in LPS-stimulated cells in the presence of DT-13 and Rk1; (**B**) relative protein expression analysis of PPARγ with respect to β-actin. The significance levels are measured with respect to untreated (UT) and LPS as depicted with lines in the graph. The experiment was conducted in triplicate, and the analysis was carried out using the average of three independent experiments; * *p* < 0.05, ** *p* < 0.01, *** *p* < 0.001, **** *p* < 0.0001.

The cells stimulated with LPS showed a decrease in PPARγ expression in comparison to untreated (UT) cells (Figure 1), which is aligned with the already known role of PPARγ in inflammation. Cells pre-treated with DT-13, Rk1 and dexamethasone alone showed an increase in PPARγ expression in comparison to cells treated with LPS alone but lower than that of untreated cells. This could be attributed to an unknown effect of DMSO. However, in LPS-stimulated cells, we observed that the LPS-induced inhibition of PPARγ protein was reversed with DT-13, Rk1 and dexamethasone pretreatments. These results depict that the addition of DT-13 regulates the expression of PPARγ and its target gene NFκB.

### 3.2. HEK293FT Cells as a Model System to Screen the Ligands of PPARγ

To further understand the effect of DT-13 on PPARγ-mediated transactivation, we used a HEK293FT cell-based transfection model. The HEK transfection model system is a standardized cell-based in vitro assay used to screen the agonists of PPARγ [28,29]. Moreover, HEK293FT cells are easy to transfect, with high transfection efficiency [30], rapidly growing and robust [31], and are also a preferred choice for expression of membrane proteins to study structure and function [32]. Cells were transfected with the pcDNA-PPARγ plasmid for the PPARγ protein and PPRE X3-TK-luc containing a PPARγ response element (PPRE) conjugated with a luciferase gene as a reporter. For the transfection reference control, pRL-SV40 was used.

**Figure 2:**
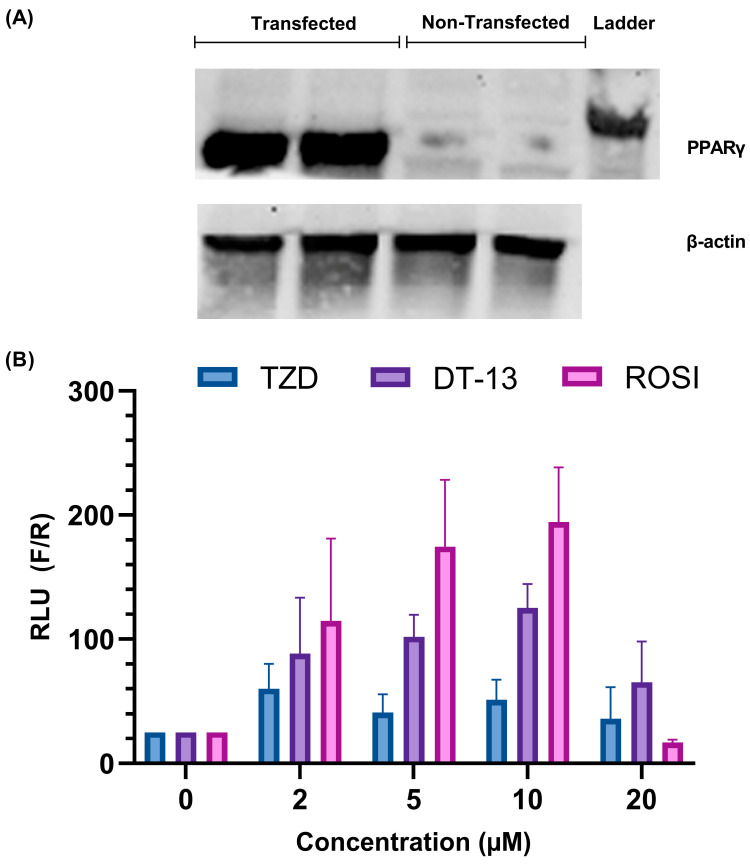
Establishment of cell-based screening of PPARγ ligands using HEK 293FT cells: (**A**) overexpression analysis of the PPARγ protein in HEK293 FT cells with the help of Western blotting; (**B**) validation of the working model of the in vitro PPARγ ligands screening assay. A dose-dependent increase in luciferase activity is observed in rosiglitazone- and troglitazone-treated cells overexpressed with the PPARγ gene. The images are representative of experiments carried out in triplicate, expressed as mean ± SD.

The overexpression of PPARγ was confirmed by Western blot as shown in Figure 2A. After transfection, the cells were induced with the ligands of PPARγ, namely rosiglitazone and troglitazone, and then luciferase activity was observed. According to the experimental setup, a positive binding of the compound to the receptor activates the PPARγ receptor, which in turn binds to the PPRE. This association leads to the active transcription of the luciferase gene. Increased expression of the luciferase gene and therefore luminescence is marked as an activation of the ligand-dependent PPARγ-mediated gene regulation.

In the presence of troglitazone (a synthetic ligand of PPARγ), the luciferase activity of the cells was higher at 2 µM than in untreated cells (Figure 2B). At higher concentrations, the activity was lower compared to 2 µM with no recognizable trend. For rosiglitazone, an increase in luciferase activity was observed until 10 µM concentration (Figure 2B). A sudden decrease in activity was observed at a ligand concentration of 20 µM. Such bell-shaped results in dose–response curves are often observed in compounds that have the tendency to self-aggregate, but it could also occur because of increased stress on cells in addition to transfection with three plasmids (see discussion). The results confirm the capability of the cell-based transfection model system to detect the ligand-mediated activation of PPARγ.

Similarly, we also tested the activity of DT-13 in comparison to the other ligands. We observed an increase in luciferase activity of the cells from 2 µM to 10 µM concentration and a subsequent decrease at 20 µM, similar to rosiglitazone, but less pronounced (Figure 2B). This result indeed marked the preliminary confirmation of the association of DT-13 in the activation of PPARγ-mediated gene regulation.

### 3.3. Saponins Activate PPARγ In Vitro

**Figure 3:**
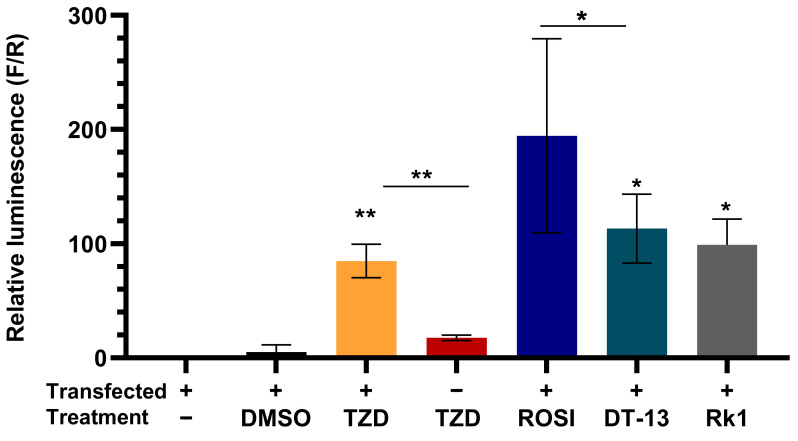
DT-13 and Rk1 induce ligand activation of PPARγ-mediated gene transcription. Graphical representation of luciferase activity of PPARγ-overexpressing cells in the presence of DT-13 and Rk1 in comparison to troglitazone-treated cells. A significant increase in luminescence with respect to untreated depicts the PPARγ-mediated PPRE-tk-LUC gene transcription. Data are represented as mean ± SD of three independent experiments, conducted in triplicate; * *p* < 0.05, ** *p* < 0.01.

To further confirm and compare the activation of PPARγ in the presence of saponins, we used a 10 µM concentration of DT-13, Rk1, and troglitazone for comparative analysis. An increased luciferase activity was seen in cells treated with rosiglitazone and troglitazone in comparison to untreated cells (Figure 3), whereas cells that were not transfected with PPARγ did not elicit any luminescence when treated with troglitazone, depicting the rigidness of the experimental model. With the treatment of DT-13 and Rk1, we observed a significant increase in luciferase activity of the cells, illuminating the role of saponins DT-13 and Rk1 in the activation of PPARγ and its response elements.

**Figure A2:**
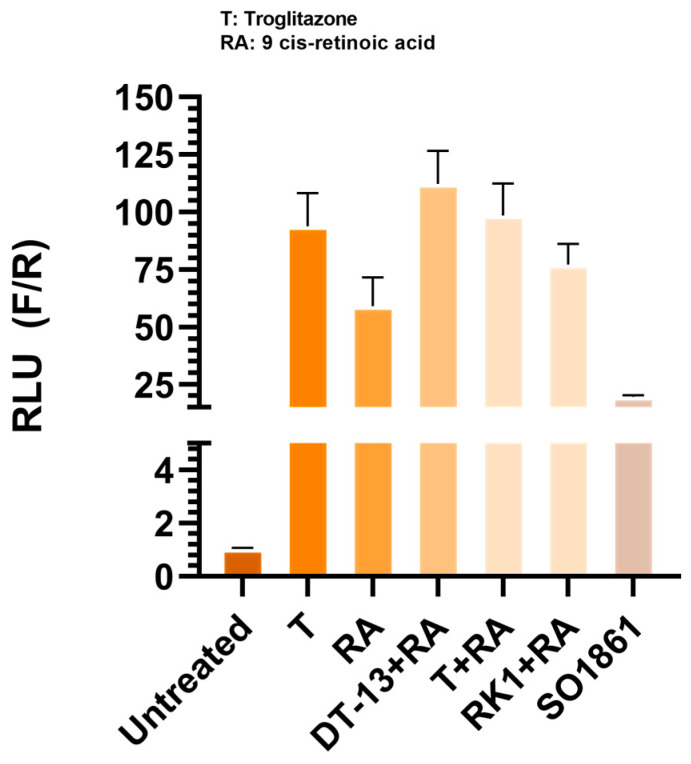
Effect of drug combination treatments in PPARγ-overexpressed cells: HEK293 FT cells expressing the PPARγ protein were treated with SO1861 and 9-cis retinoic acid (RA) in combination with troglitazone (T), DT-13 and Rk1. No significant increase in luciferase activity was observed in cells treated with SO1861 alone. The addition of RA with T did not increase the luminescence in comparison to T-alone-treated cells. Data are represented as mean ± SD of three independent experiments.

To rule out the possibility that any addition of a saponin elicits the gene expression, we induced the cells with another saponin—SO1861, which is known as an endosomal escape enhancer [33]. There are no findings on SO1861 acting as an anti-inflammatory molecule yet. We found out that SO1861 had no significant effect on the activity of the luciferase gene. This confirms the reliability of the experimental setup that not all natural saponins can induce the gene expression, and therefore a significant positive binding to the receptor is required to induce luciferase activity as a marker for PPARγ-mediated gene expression. PPARγ is known to be co-expressed as a heterodimer with retinoic acid receptor (RXR) [34], although activation of either of the receptors is required for the ligand-mediated effect [35]. Therefore, to understand the effect of the combination of both ligands, we pretreated cells with troglitazone in combination with the RXR agonist 9-cis retinoic acid (RA). We observed no difference in cells treated with troglitazone in comparison to troglitazone plus RA. Similarly, there was no significant increase in combination treatments of the saponins DT-13 and Rk1 with RA (Appendix A Figure A2). However, this could also be attributed to the overexpression of PPARγ and not the RXR protein.

### 3.4. Comparative Analysis of Binding Efficiency of PPARγ Ligands and DT-13

To determine the binding affinities of PPARγ ligands, a fluorescent polarization-based PolarScreen™ PPARγ-competitor assay kit was used. Synthetic agonists—rosiglitazone, troglitazone and PPARγ inhibitor GW9662—showed a dose-dependent decrease in fluorescence polarization, depicting the increased binding efficiency of these compounds in a dose-dependent manner. A shift in fluorescence polarization was observed at around 100 nM concentration of the natural ligand 9-hydroxyoctadecadienoic acid (9-HODE). Surprisingly, DT-13 and Rk1 did not show a major change in the polarization values. The half-maximum inhibition concentrations (IC50) equivalent to the binding affinity of the molecules were calculated as half-maximal polarization values of the compounds.

**Figure 4:**
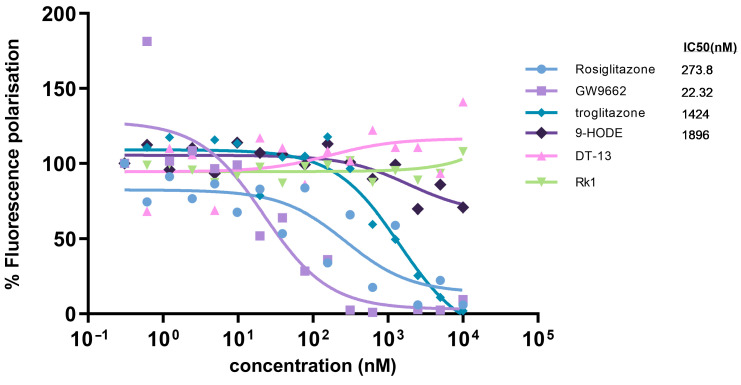
Comparative analysis of binding efficiency of PPARγ ligands using a competition assay. The binding affinity of test compounds (PPARγ ligands and saponins—DT-13 and Rk1) with the PPARγ ligand-binding domain was determined using a fluorescence polarization-based competitive binding assay. Fluorescence polarization values were converted to percent fluorescence polarization, considering the individual lowest concentration value as 100%. Curve fitting was performed using GraphPad Prism 9.0, and the values are represented as the mean of triplicate reaction wells. IC50 values are representative of the relative binding affinity of test compounds.

We observed that the IC50 values for rosiglitazone and troglitazone were 273.8 nM and 1.42 µM, respectively. The IC50 value of the PPARγ inhibitor GW9662 was 22.32 nM, and for 9-HODE, 1.8 µM. DT-13 showed an increase in polarization values at higher concentrations, and an arbitrary IC50 value was obtained. Rk1, on the other hand, appeared to not affect the polarization values and had an ambiguous IC50 value (Figure 4). The polarization values are normalized in percentage by keeping the lower concentration values as maximal polarization of 100% and calculating the individual values with respect to it.

### 3.5. In Silico Binding of DT-13 to PPARγ

**Figure 5:**
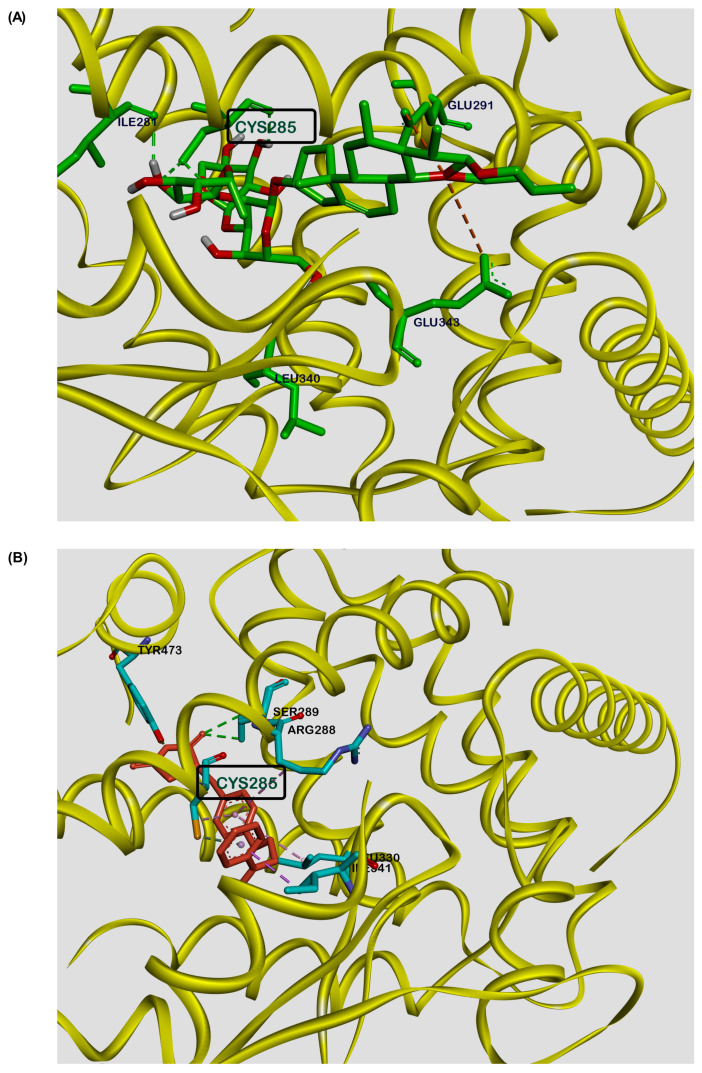
Molecular docking studies depicting a putative binding site for DT-13 at the PPARγ ligand-binding pocket. Representative image showing molecular interaction of (**A**) DT-13 and (**B**) rosiglitazone, with the ligand-binding pocket of PPARγ protein as visualized by Discovery Studio Visualizer. Both the molecules showed similar binding interactions at Cys285.

To study the possible binding interaction of DT-13 with the PPARγ protein, molecular docking simulations were performed using Autodoc Vina [36]. Docking calculations showed the possible binding of DT-13 at the binding site of the PPARγ protein, like that of rosiglitazone (Figure 5). The best conformation of DT-13 had a binding energy of –5.11 kcal/mol, indicating moderate potential binding affinity for PPARγ. The glycosidic part of DT-13 showed three interactions via hydrogen bonding (Ile-281, Cys-285, Leu-340) and two electrostatic interactions (Glu-291, Glu-343) with the steroidal part. Similar interactions were observed for the synthetic ligand rosiglitazone in the co-crystal structure. It forms two hydrogen bond interactions (Tyr-473, Ser-289) with the polar head, two hydrophobic interactions (Leu-330, Arg-288) and one hydrogen bond (Cys-285) at the aromatic central ring, and another two hydrophobic interactions (Cys-285, Ile-341) at the lipophilic tail (Figure 5). The binding of the molecule to the Cys-285 amino acid residue is known to be important to exert PPARγ-induced transactivation [29]. Interestingly, DT-13 and rosiglitazone both showed strong hydrogen bond interactions with cysteine at position 285 of PPARγ. These results suggest the moderate putative binding of DT-13 with PPARγ and support the findings of transfection experiments depicting DT-13 as a ligand for PPARγ to mediate transactivation of genes.

## 4. Discussion

Many new drug therapeutics are widely based on nature-derived compounds [37]. They are preferred over synthetic drugs as they are easily absorbed by the body with reduced side effects [38,39]. DT-13, a steroidal saponin, is obtained from the roots of *Liriope muscari* L. H. Bailey and is known for its medicinal benefits. In our previous findings, we confirmed that DT-13 inhibits translocation of activated NFκB to the nucleus and thereby prevents the transcription of proinflammatory genes in LPS-stimulated RAW264.7 macrophages [18]. However, there was no evidence of DT-13 binding to nuclear receptors. There is evidence that saponins are ligands for nuclear receptors [40,41]. Therefore, to advance the existing knowledge on pharmacological effects of DT-13, the current study aimed to investigate PPARγ as a therapeutic target of DT-13 in vitro. Since members of nuclear receptors are known to be involved in immunity and inflammation via transrepressing the inflammatory genes [20,42], we explored the possibility of DT-13 and Rk1 (ginseng saponin) initiating the ligand-dependent PPARγ activation. LPS is known to decrease the expression of PPARγ protein in vitro [43]. In contrast to the untreated (UT) cells, Western blot analysis showed reduced expression of PPARγ in cells treated with LPS alone. DT-13 and Rk1 induced expression of PPARγ in LPS-stimulated RAW264.7 macrophages (Figure 1A). This depicts the involvement of DT-13 and Rk1 in the expression of PPARγ in LPS-stimulated cells. PPARγ is mostly present in macrophages, adipocytes and the colon [44]. Having identified the effect of DT-13 on PPARγ, future studies should look at whether the effects also occur in such other cells and, if so, how they might differ. In this study, we used a non-immune cell-based in vitro model to overexpress the PPARγ receptor and to analyze pathway-specific activation. This model is described and already used in other studies to screen PPARγ ligands [28] and to study the immunomodulatory pathways [45]. Moreover, cells of myeloid origin, such as RAW264.7 cells, are programmed to respond to foreign genes so that these immune reactions triggered overlap with the effects of the gene product [46].

With the help of a HEK transfection model system to screen the ligands in vitro [28], we observed an increase in luciferase activity in cells treated with DT-13, depicting the ligand-dependent PPARγ-mediated gene activation (Figure 2B). We standardized the model using the known PPARγ ligand troglitazone and checked its efficacy with rosiglitazone. However, a decreased luciferase activity observed at 20 µM rosiglitazone treatment (Figure 2B) could be the result of high transfection stress in addition to cytotoxicity [47].

Additionally, our results demonstrated that DT-13 and Rk1 significantly increased the expression of the reporter luciferase gene corresponding to the ligand-mediated activation of PPARγ in comparison to troglitazone and rosiglitazone (Figure 3). Another nature-derived saponin—SO1861, known for its endosomal escape-enhancing property [33]—was used to evaluate the efficacy of the cell-based ligand screening model system. Only minimal luciferase activity was observed in cells treated with SO1861 (Appendix A Figure A2), demonstrating that not every natural compound can induce the gene activation unless binding to PPARγ. We further evaluated the binding efficiency of the ligands of PPARγ—rosiglitazone, troglitazone and 9-HODE—and the inhibitor GW9662 and saponins DT-13 and Rk1 using a competitive fluorescent polarization-based screening kit. We observed a dose–response curve for the known ligands and inhibitors of the PPARγ protein. The IC50 values for rosiglitazone and troglitazone were 0.27 µM and 1.4 µM, respectively (Figure 4). The fluorescence polarization observed ranged between 100 and 150 mP, which depicts a good range for dose–response curves [48]. Interestingly, DT-13 and Rk1 showed an increase in the fluorescence intensities at the parallel excitation plane than that of emission from the perpendicular plane that resulted in higher fluorescence polarization values (mP). This result could be due to background interference [49]. Further cleanup and enrichment of the saponins could have reduced the effect, but it also increases the risk of loss of the molecule due to limited solubility and micellar formation [50]. However, in addition to the robust cell-based ligand screening method to identify the ligands of PPARγ, a cost-effective cell-free assay might serve as a quick analysis for the identification of new ligands [51]. Previous in silico studies identified possible binding interactions for various saponins in the ligand-binding domain of PPARγ [26]. Our findings showed DT-13 docked into the ligand-binding domain of PPARγ similar to that of rosiglitazone. According to the docking calculations, DT-13 showed a moderate binding affinity of –5.11 kcal/mol. Both the molecules showed strong hydrogen binding with Cys-285, which is important for the transactivation effect of PPARγ (Figure 5). For rosiglitazone, Zhou et al. showed inhibition of LPS-induced inflammatory responses in RAW264.7 macrophage cells, which was dependent on PPARγ activation and NF-κB suppression [52]. Although only theoretically proven by our study, it can be supposed that both substances (rosiglitazone and DT-13) act analogously. The observation that DT-13 is not able to replace the fluoromone only indicates lower affinity, but it neither excludes binding nor functionality. Nevertheless, the fluorescence polarization results can also point towards a possibility of an alternate binding [53,54]. Alternate site binding likely contributes to PPARγ hyperactivation in vivo, perhaps explaining why PPARγ full and partial or weak agonists display similar adverse effects [53]. Other binding approaches, such as surface plasmon resonance, could be used to experimentally show binding of DT-13 to PPARγ but cannot reveal the binding site. Apparently, the interactions observed in this study were similar to most of the PPARγ ligands screened virtually [55]. The focus of this study was to understand the anti-inflammatory mechanism of DT-13 mediated by PPARγ activation, which is demonstrated using a HEK293FT transfection model. The results were validated using different controls, including rosiglitazone, troglitazone, dexamethasone and saponin SO1861. However, it cannot be excluded that the effect of DT-13 is tissue specific. This can be explored by investigating other cell lines from different tissues and in vivo studies. In addition, RAW264.7 cells may not be suitable for all types of drug screening assays, and their response to some drugs may differ from that of primary macrophages. Taken together, our results illustrated that anti-inflammatory DT-13 is involved in ligand-mediated activation of PPARγ to regulate proinflammatory genes.

## 5. Conclusions

The current study aimed at exploring the effects of anti-inflammatory DT-13 on the transactivation properties of PPARγ in vitro. We successfully investigated and showed that DT-13 is involved in ligand-dependent PPARγ-mediated activation of PPARγ response elements via standardized luciferase assay in HEK293 cells. We also compared the effect of known PPARγ ligands—rosiglitazone and troglitazone—with saponins DT-13 and Rk1. We observed that DT-13 showed increased activation of PPARγ response elements in comparison to rosiglitazone and troglitazone. The binding assays using the fluorescence polarization technique did not show the binding of DT-13 to the PPARγ, but they pointed out an alternate binding. With the help of docking studies, we showed that DT-13 has a putative binding site similar to that of rosiglitazone with a moderate binding energy. We also found that it binds to the amino acid cysteine at position 285, which is critical for the activity of PPARγ to mediate transactivation of genes. Together our results conclude that DT-13 activates PPARγ response elements in a PPARγ ligand-dependent manner in vitro, and this pathway of transactivation could be involved in the anti-inflammatory properties of DT-13 as published earlier by our group. This study is important to provide the fundamental data for the preclinical studies needed to develop DT-13-based targeted anti-inflammatory drug molecules.

## Data Availability

This study does not contain any experimentally gained structural data, sequence data, functional genomics or genetics data. Computational models will be available at the Protein Data Bank in Europe (PDBe).
